# Risk factors at icu admission for multi-resistant acinetobacter baumannii colonization and infection and its prediction capability

**DOI:** 10.1186/2197-425X-3-S1-A127

**Published:** 2015-10-01

**Authors:** R Vara Arlanzón, S Puerto Corrales, F Callejo-Torre, JM Eiros Bouza, MJ Coma del Corral, M Martínez Barrios, ME Perea Rodríguez, M Montero Baladía, S Ossa Echeverri, S Calvo Simal, M del Valle Ortíz, O Badallo Arévalo, C Carbajales Pérez, JA Fernández Ratero, ENVIN-HELICS Study Group

**Affiliations:** Hospital Universitario de Burgos, Intensive Care, Burgos, Spain; Hospital Clínico Universitario de Valladolid, Microbiology, Valladolid Spain; Hospital Universitario de Burgos, Burgos, Spain

## Introduction

Predicting colonization/infection by *Acinetobacter baumannii* (C-I MRAb) at ICU admission allows optimization of infection control measures and empirical antibiotic treatment.

## Objectives

To describe risk factors for multirresistant *Acinetobacter baumannii* (defined as resistant to carbapenem) colonization and infection at ICU admission, centered on those that are easy to obtain at admission, without needing access to clinical records, not always avaible at ICU admission. We also study the predictive capability of a model developed with those risk factors.

## Methods

Retrospective analysis of data collected prospectively from 16950 patients admitted consecutively (stay > 24h) in 151 Spanish ICU participating in ENVIN (National Surveillance Study of Nosocomial Infections in ICU) registry during the period of April-June of 2010. Univariable and multivariable analysis was performed with the next variables: age, gender, illness, origin place, urgent surgery, immunodeficiency, being immunosuppressed, neutropenic patients and skin infections (surgical site -superficial and deep- and skin-soft tissue infections).

## Results

Overall, 44 patients with C-I MRAb were detected in the study period. In the multivariable logistic regression analysis gender male (2,99(1,32-6,77)0,008), to have medical illness (3,16(1,42-7,02)0,005), trauma critical patient (4,74(1,44-15,56)0,01), admitted from hospital ward (6,21( 2,49-15,47) < 0,001), or from other ICU (12,92(3,87-43,15) < 0,001), or from a long-term care center (11,95(1,38-102,97)0,02), being immunosuppressed (4,52 (2,2-9,27) < 0,001) and to have skin-soft tissue infection (14,07 (4,26- 46,46) < 0,001) were independent risk factors for C-I MRAb at ICU admission. The resultant predictive model with these variables showed: AUC-ROC 0,85; 95% CI (0,79-0,90) and p-value on the Hosmer-Lemeshow of 0,89.

## Conclusions

The risk of being C-I MRAb at ICU admission is 3 times greater in male and medical patients, 4 times in immunosuppressed or in trauma critical patients, 6 times in those admitted from hospital ward, 12 times in patients admitted from other ICU or from a long-term care center and 14 times in those that have skin-soft tissue infection. Our predictive model showed good discrimination but these results are not good enough for ICU setting, being aware of the impact of the false negative results. Risk factors described should be considered for future studies.
